# Non-invasive estimation of left ventricular chamber stiffness using cardiovascular magnetic resonance and echocardiography

**DOI:** 10.1016/j.jocmr.2025.101849

**Published:** 2025-01-31

**Authors:** Ida Marie Hauge-Iversen, Einar S. Nordén, Arne Olav Melleby, Linn Espeland, Lili Zhang, Ivar Sjaastad, Emil Knut Stenersen Espe

**Affiliations:** a Institute for Experimental Medical Research, Oslo University Hospital and University of Oslo, Oslo, Norway; b Institute of Basic Medical Sciences, University of Oslo, Oslo, Norway

**Keywords:** Imaging biomarkers, Cardiac imaging, Preclinical research, Diastolic function, Chamber stiffness

## Abstract

**Background:**

Preclinical studies exploring the underlying mechanisms of elevated left ventricular (LV) chamber stiffness play a crucial role in developing new therapeutic strategies. However, there is a lack of systematic evaluation of imaging biomarkers of diastolic function against gold standard assessment of LV chamber stiffness in rodents. Therefore, we aimed to evaluate imaging biomarkers of diastolic function from cardiovascular magnetic resonance (CMR) and echocardiography in predicting the slope of the end-diastolic pressure-volume relationship (EDPVR) in rats.

**Methods:**

Sprague Dawley rats with varying degrees of myocardial stiffness induced by aortic constriction (n=38) and healthy controls (n=9) underwent echocardiography and CMR at approximately 13 weeks post-operation. Imaging biomarkers of diastolic function were evaluated for their ability to predict the EDPVR slope from pressure-volume recordings using regression analysis and receiver operating characteristics analysis.

**Results:**

Both CMR and echocardiographic imaging biomarkers, in particular those related to the left atrium and mitral flow, were able to predict the EDPVR slope in a rat model with varying stiffness. From CMR, native T1 values, peak early diastolic longitudinal strain rate (SRe(long)) and E/SRe(long), left atrial (LA) ejection fraction, isovolumetric relaxation time (IVRT), E/A and peak LA strain, correlated best with the EDPVR slope (|r|=0.54–0.72). From echocardiography, E/A, E, LA diameter, e’/a’, E/SRe(long) and IVRT correlated with the EDPVR slope (|r|=0.49–0.67), while E/e’, e’ and E-wave deceleration time demonstrated poor correlation (|r|=0.17–0.27). Receiver operating characteristics analysis indicated better performance of CMR imaging biomarkers than echocardiography in predicting increased EDPVR slope.

**Conclusions:**

Several diastolic imaging biomarkers commonly employed in preclinical studies have poor ability to predict cardiac chamber stiffness. Our study identifies several imaging biomarkers obtained from both echocardiography and CMR that are able to estimate LV chamber stiffness non-invasively, providing an important tool for future mechanistic research on myocardial stiffness.

## Graphical Abstract

**Figure:**
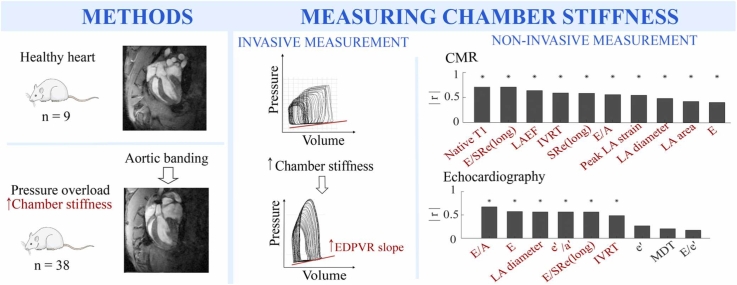
**Measuring chamber stiffness** Increased afterload has shown to lead to diastolic dysfunction and increased chamber stiffness. Chamber stiffness can invasively be assessed by measuring the slope of the end-diastolic pressure-volume relationship (EDPVR) from pressure-volume catheterization. Imaging biomarkers from cardiovascular magnetic resonance (CMR) and echocardiography correlated with the EDPVR slope, accomplishing non-invasive estimation of chamber stiffness. r is the value of Pearson’s correlation coefficient. *p<0.05, correlation against the EDPVR slope. EF: Ejection fraction, MDT: Mitral deceleration time, IVRT: Isovolumetric relaxation time, LA: Left atrial, SR: Strain rate.

## 1 Introduction

Increased left ventricular (LV) chamber stiffness is a hallmark of several forms of pathological cardiac remodelling, including that of hypertension, aortic stenosis, and cardiac fibrosis [1], [2], [3]. This causes diastolic dysfunction and may lead to heart failure, with subsequent high morbidity and mortality [4], [5], [6]. While historically emphasis has been placed on estimating cardiac filling pressures, recent publications have advocated the importance of estimating cardiac stiffness in the era of precision medicine [7]. Patients with increased cardiac stiffness have been shown to respond differently to treatment [8]. Consequently, development of therapy targeting the underlying mechanisms of increased LV chamber stiffness is of the utmost importance, and preclinical trials are vital in such endeavours. To facilitate such investigations, accurate estimations of cardiac stiffness in preclinical trials are of great importance.

A gold standard for assessing LV chamber stiffness in both humans and rodents is measuring the slope of the end-diastolic pressure-volume relationship (EDPVR) [6], [9], [10]. This measurement is derived from pressure-volume (PV) recordings obtained from LV catheterization [11]. When normalized to volume, similar EDPVR slopes are found in rat, dog, and human hearts indicating a general applicability across species [12]. Measuring EDPVR relies on highly invasive techniques with extensive technical requirements and limited possibilities for longitudinal studies [11]. Therefore, imaging biomarkers that can predict the EDPVR slope would be of great utility in preclinical studies. To our knowledge, systematic validation of imaging biomarkers against the EDPVR slope has not been done in small animals.

Several imaging biomarkers of diastolic function may reflect the EDPVR slope. As cardiac chamber stiffness increases, LV filling pressure elevates [13]. The left atrium is directly affected by the LV diastolic pressure, also during late diastole, potentially making left atrial (LA) size and function parameters sensitive markers of alterations in the EDPVR slope. Increased LV stiffness could also impact blood flow velocities during passive and active filling, as reflected by early- and late LV mitral inflow velocities (E and A) and mitral E-wave deceleration time (MDT). Increased chamber stiffness furthermore affects the ventricular relaxation pattern, potentially decreasing early LV annular velocity (e’), late LV annular velocity (a’) and peak early diastolic longitudinal strain rate (SRe(long)). Alterations in relaxation patterns and pressure changes also influence timing of valvular events, which consequently affect the isovolumetric relaxation time (IVRT) [13]. Additionally, myocardial remodelling, such as increased fibrotic deposition, can influence both chamber stiffness and native T1 values of the tissue from cardiac magnetic resonance (CMR) [14].

Imaging biomarker of diastolic function can be assessed using both CMR and echocardiography. Echocardiography is the more easily available and commonly used imaging modality to assess diastolic properties in both humans and rodents [15], [16]. However, CMR has unmatched geometric control and is the gold standard for assessing ventricular and atrial volumes. Furthermore, MRI allows access to more advanced techniques, such as T1 mapping [17], [18].

In this study, our aim was to evaluate the ability of CMR and echocardiography to predict the EDPVR slope in a well-established animal model of graded LV chamber stiffness. We utilized a rat model of aortic banding as this model of cardiac pressure overload induces concentric hypertrophy and cardiac fibrosis, and where LV chamber stiffness is one of the main drivers of diastolic dysfunction. It is a robust animal model with known alteration in LV chamber stiffness [19], and is widely used to investigate mechanisms of cardiac fibrosis [20].

## 2 Methods

### 2.1 Ethics

Animal experiments were approved by the Norwegian National Animal Research Committee (approval #20208), which conforms to the National Institutes of Health guidelines (NIH Publication No. 85–23, Revised 1996).

### 2.2 Study design

We utilized male Sprague Dawley rats (Janvier Labs, Le Genest-Saint-Isle, France), weighing approximately 100 g (∼1 month old). The animals were housed 2–3 animals per cage, maintained under a 12-hour light-dark cycle, and provided ad libitum access to food and water. After aortic banding or sham surgery, at approximately 13 weeks post-surgery, a total of 47 rats (controls: n=9; aortic banding: n=38) underwent pressure-volume catheterization and were sacrificed (median of 92 days, interquartile range: 89–93 days). CMR and echocardiography were acquired at a median of 3 days before catheterization (CMR range: 2–5 days, echocardiography interquartile range: 2–7 days). CMR and echocardiography were performed at least one day prior to cardiac catheterization to allow recovery from prolonged anaesthesia [21]. Animals were sacrificed directly after PV catheterization and heart tissue was weighed and a left midventricular section was collected for histology. Tibia length was measured to detect stunting of growth in aortic banded animals. One aortic banded animal was excluded due to poor PV recording. All analyses, including CMR, echocardiographic, and PV analysis, were performed by a single operator blinded to information from other analyses and surgical intervention.

### 2.3 O-ring aortic banding

Aortic banding was performed to induce cardiac pressure overload, as previously described by our group [19]. Only male rats were included due to lack of validation of the model in female rodents. Anaesthesia was induced with 4–5% isoflurane in O_2_. After intubation the animals were placed on a dedicated small-animal ventilator (VentElite, Harvard apparatus, Holliston, Massachusetts) and anaesthesia maintained with 2% isoflurane in O_2_. The thoracic cavity was opened, and an O-ring (Polymax, Bordon, United Kingdom) with internal diameter of either 1.5 mm (n=6), 1.3 mm (n=27) or 1.07 mm (n=5) was placed around the ascending aorta. Sham-operated animals (n=9) underwent the same surgical procedure, except for the insertion of an O-ring, and served as controls. Using different O-rings, in combination with controls, we aimed to induce a wide range of LV stiffness, from normal to high levels. O-rings with an internal diameter of 1.3 mm were used to induce a wide distribution of LV stiffness (based on unpublished data). Additional O-rings with internal diameter of 1.5 mm and 1.07 mm were used to supplement the lower and higher ranges of LV stiffness, respectively. The controls provided animals with a normal range of LV stiffness.

Local and systemic analgesia were administered prior to surgery, with the first 18 animals receiving 0.6 mg bupivacaine at the incision site prior to surgery, and a subcutaneous injection of 0.05 mg/kg buprenorphine ½ hour before, and 8, 16 and 24 h after surgery. Following updated institutional procedures, the subsequent 28 animals received 0.4 mg/kg buprenorphine by oral gavage 1 h before and 24 h after surgery in addition to subcutaneous bupivacaine [22].

### 2.4 CMR examination

Anaesthesia was induced with ∼4% and maintained with 1.5–2% isoflurane in O_2_. CMR was conducted using a 9.4 T magnet (Agilent Technologies, Palo Alto, California) interfaced to Avance Neo console (Bruker Biospin, Ettlingen, Germany). Animals were monitored throughout the entire examination by electrocardiogram (ECG), respiration rate and body temperature. Temperature was regulated using heated air to maintain a body temperature of 37 °C. Cine CMR, tissue phase mapping, mitral flow velocity and native T1 mapping were recorded allowing analysis of new and commonly used parameters for assessing diastolic dysfunction [17].

Cine CMR examination included a four-chamber and a two-chamber long-axis view and a stack of short-axis slices (14–18 slices) covering the entire left ventricle and left atrium. Cine long-axis were acquired in a fully sampled manner, while the stack of short-axis slices was acquired using compressed sensing with 4x undersampling and reconstructed as previously described [23]. Key acquisition parameters for Cine CMR were: slice thickness = 1.5 mm, matrix = 128 ×128 (for fully sampled) or 128 ×32 (for compressed sensing), field of view (FOV) = 45 mm × 45 mm, repetition time (TR) = 4.17 ms, echo time (TE) = 2.12 ms, flip angle = 12° (for fully sampled) flip angle = 15° (for compressed sensing), signal averaging = 2–3, total cine CMR acquisition time = ∼20 min.

Tissue phase mapping was examined in a four-chamber long-axis view and was acquired with a radiofrequency-spoiled black-blood gradient echo nine-point phase contrast-CMR protocol [24], using a compressed sensing technique with 4x undersampling as previously described [23]. Briefly, a pair of bipolar motion encoding gradients were used to encode the motion of the tissue into the signal phase. The data was acquired in a non-interleaved manner, so the temporal resolution of the dataset was equal to the TR. Key acquisition parameters for CMR long-axis tissue phase mapping: matrix = 128 ×32, velocity encoding = 20 cm/s, FOV = 45 mm × 45 mm, TR = 4.17 ms and TE = 1.98 ms, flip angle = 10°, signal averaging = 1, slice thickness = 1.5 mm, and acquisition time = ∼4 min per slice.

Mitral flow recordings included three slices, one placed perpendicular to the blood flow at the tip of the mitral leaflets during mitral valve opening, and two additional slices were acquired with a parallel shift of 0.5 mm in the apical and basal direction from the position of the first slice. Mitral flow was acquired with the same radiofrequency-spoiled gradient echo nine-point phase contrast-CMR protocol with compressed sensing, but without black blood preparation, as previously described [25]. Key acquisition parameters for CMR mitral flow: matrix = 128 ×32, velocity encoding = 200–300 cm/s, FOV= 45 mm × 45 mm, TR = 4.17 ms and TE = 1.98 ms, flip angle = 10°, signal averaging = 1, slice thickness = 1 mm, and acquisition time = ∼4 min per slice.

The native T1 mapping examination included three short-axis slices, recorded at the basal, midventricular and apical myocardium levels. Native T1 mapping included a Look-Locker sequence with a spoiled, multi-slice, segmented gradient-echo readout. Key acquisition parameters for native T1 mapping were slice thickness = 1.5 mm, matrix = 128 ×64 (zero filled to 128 ×128), FOV = 45 mm × 45 mm, true TR = 12.5 s, TE = 2.41 ms, flip angle = 8°, segment size = 4, echo images = 40, acquisition time = ∼4 min. Native T1 mapping was not performed if stable ECG triggering was not achieved during scanning. Good quality images were achieved in 33 out of 47 animals and analysed as a separate sub-study.

### 2.5 CMR analysis

CINE CMR was analysed using Segment v3.2 R8456 (Medviso AB, Lund, Sweden) [26], whereas tissue phase mapping, mitral flow and native T1 mapping were analysed using Matlab R2018 (The MathWorks, Inc., Natick, Massachusetts). Compressed sensing data were zero-filled and reconstructed with a temporal Fourier compressed sensing reconstruction algorithm using Matlab R2018a as previously described [23]. Images with poor image quality were excluded from analysis.

From cine CMR short-axis slices covering the whole left ventricle and left atrium, maximum and minimum volumes were segmented and used to assess LV end-diastolic volume, LV stroke volume, LV EF and LA EF. LA diameter, LA area, LA strain and IVRT were measured from the four-chamber long-axis slice. IVRT was calculated by counting the number of time frames between aortic valve closure and mitral valve opening and multiplying with the TR.

LA strain analysis was performed by feature tracking using Segment v3.2 R8456 as previously described [26], [27]. The endocardium of the left atrium was segmented, and LA strain was calculated in a semi-automatic manner for every time point of the heart cycle. Peak LA strain was defined as the maximum LA strain during the cardiac cycle.

Reconstructed tissue phase mapping images of the four-chamber long-axis view were semi-automatically segmented using a previously described software in Matlab R2018 [24], and used to calculate longitudinal strain rates (SR). SRe(long) was defined as the peak of the strain rate curve in early diastole.

In the mitral flow recordings, the region of interest included the blood flow through the mitral valve segmented from the early diastolic time frame (defined from the velocity recording). The velocities in the region of interest were plotted, and E and A were decided from the maximum velocity detected in the three recorded slices.

Before the calculation of native T1 values all the images with motion artifact were discarded. LV myocardium on all three slices were segmented and fitted using least squares to construct the inversion recovery curve. The native T1 values were calculated as the average from all three slices.

### 2.6 Echocardiographic examination and analysis

Anaesthesia was induced with ∼4% and maintained with 1.5–2% isoflurane in O_2_. The animals were placed on a heating pad to maintain body temperature and ECG was continuously recorded. Transthoracic echocardiography was conducted using a Vevo 3100 (Fujifilm VisualSonics, Toronto, Canada). LV long-axis and short-axis B-mode, midventricular and basal M-mode from long-axis, pulsed wave Doppler of diastolic mitral flow and tissue Doppler at the posterior wall, were recorded [28]. This allowed assessment of the most commonly used parameters for assessing diastolic dysfunction [29].

Images were analysed in Vevo Lab 3.2.0 (Fujifilm VisualSonics, Toronto, Canada). Images with poor image quality were excluded from analysis. E´ and a’ were measured in the short-axis plane. Doppler recordings of mitral inflow from an apical two-chamber view provided E, A and MDT. IVRT was calculated from pulse wave Doppler of the aorta outflow and mitral inflow in two separate recordings, by subtracting the time from the ECG-determined R-peak to the end of aorta outflow from the time from R-peak to the start of mitral inflow. LA diameter was measured from the long-axis basal M-mode. SRe(long) was measured by speckle tracking from long-axis images using as the peak in early diastole from the strain rate curve gained from speckle tracking in a long-axis view using Vevo Strain (Fujifilm VisualSonics, Toronto, Canada).

### 2.7 Pressure-volume catheterization and analysis

For pressure-volume catheterization anaesthesia was induced with 3% and maintained with 2.5% isoflurane in O_2_. After intubation and ventilation previously described, a transverse incision was made at the subcostal level, and the diaphragm was opened. A 25 G needle was used to stab through the LV apex, and a 2 F pressure-conductance catheter (Millar Instruments, Houston, Texas) was inserted into the LV cavity through the stab. Recordings were made both during steady state and preload variation. To obtain preload reduction, the inferior vena cava was compressed immediately superior to the diaphragm using a cotton-swab tip.

EDPVR slope and tau were obtained using LabChart 8 (ADInstruments, Dunedin, New Zealand). Volume data were calibrated to the stroke volume obtained from CMR before the calculation of EDPVR. Pressure-volume loops covering the gradual reduction of preload were selected, and the EDPVR slope was automatically calculated in the software as the slope of the linear fit to the end-diastolic time point for all selected pressure-volume loops [30]. Pressure-volume loops were visually inspected, and loops from ectopic beats were manually excluded before analysis. Tau was identified as the time constant of the exponential pressure decay during the isovolumic relaxation phase, quantifying how fast the ventricular pressure falls in early diastole, reflecting cardiac relaxation [30].

### 2.8 Histological preparation and analysis

A left midventricular section fixated in 10% formaldehyde was paraffin embedded and sectioned at 6 µm using a microtome (Epredia HM 355S, Thermo Fisher Scientific Inc., Waltman, Massachusetts). Two sections per heart were stained with Masson’s Trichrome automatic staining machine (Ventana Medical Systems, Inc., Tucson, Arizona). High resolution images were acquired in an automated slide scanner system (Axio Scan Z1, Carl Zeiss Microscopy, Munich, Germany). Representative images are represented in Supplementary Figure 1. The amount of fibrosis was quantified using an open-source software QuPath version 0.4.3 [31]. Fibrosis was reported as the average of two slices.

### 2.9 Statistics

Characteristics data are presented as median (interquartile range). Medians were compared using Kruskal-Wallis test with multiple comparison adjustment where appropriate. A p<0.05 was considered statistically significant. Pearson’s correlation was used to investigate the relationship between to variables. A correlation was considered low if the correlation coefficient (r) was less than 0.5, moderate if between 0.5 and 0.69 and high if it was 0.7 or higher. The data were checked visually for equal variance by plotting residuals against fitted values and for normal distribution of residuals by QQ-plots (results are shown in Supplementary Figures 2 and 3). Least-squares fit was used for multivariate regression. A stepwise multivariate regression included all imaging biomarkers within a modality that showed a statistically significant correlation with the EDPVR slope from Pearson’s correlation. In a stepwise fashion, the imaging biomarker with the least predictive value was excluded, until only imaging biomarkers with statistically significant independent predictive value of the EDPVR slope remained. Independent predictive value was defined as having p<0.05 after stepwise multivariate regression analysis. These imaging biomarkers were used to form a composite score with the β-value from the regression analysis. Receiver operating characteristics analysis was used to determine parameter ability to detect increased EDPVR slope. Normal EDPVR slope was defined as the 95% confidence interval range of the mean of control animals. Data from receiver operating characteristic analysis are presented as area under the curve (AUC) with 95% confidence interval, where confidence intervals were truncated above 1. Statistical analyses were performed using Matlab R2022b.

## 3 Results

### 3.1 Main characteristics

No statistically significant differences were found in body size between aortic banded and control rats based on body weight or tibia length. Aortic banded rats exhibited increased EDPVR slope, levels of fibrosis, LV weight, and heart weight, and lower tau compared to controls (Table 1). All quantitative values of CMR and echocardiographic imaging biomarkers for sham operated controls, and O-rings with inner diameters of 1.5 mm, 1.3 mm and 1.07 mm can be found as median value (interquartile range) in Supplementary Tables 1 and 2.

**Table 1:** Characteristics of rat model.

	**Controls (n=9)**	**Aortic banded (n=37)**
**EDPVR slope (mmHg/ml)**	0.95 (0.94−1.02)	2.12 (1.64−2.82)*
**Tau (ms)**	16.1 (14.0−17.3)	12.3 (11.0−14.0)*
**LV EDP (mmHg)**	8.60 (6.50−9.13)	9.59 (7.42−15.64)
**-dP/dt min (mmHg/s)**	4818 (3952−5745)	5354 (4303−6353)
**Fibrosis (%)**	1.66 (1.44−2.16)	2.53 (1.93−3.28)*
**LV weight (g)**	1.06 (1.04−1.10)	1.54 (1.39−1.67)*
**RV weight (g)**	0.25 (0.24−0.30)	0.28 (0.26−0.37)
**LV EDV (ml)**	0.75 (0.68−0.80)	0.70 (0.64−0.79)
**LV SV (ml)**	0.48 (0.42−0.50)	0.47 (0.39−0.51)
**LV EF (%)**	62.8 (60.2−68.3)	66.1 (59.5−71.0)
**Lung weight (g)**	1.93 (1.73−2.00)	2.02 (1.80−2.46)
**Heart weight (g)**	1.45 (1.36−1.51)	1.91 (1.74−2.27)*
**Body weight (g)**	589 (583−637)	621 (596−639)
**Tibia length (mm)**	43.0 (42.0−44.7)	43.4 (42.7−45.3)

Data presented as median (interquartile range).

*EDP* end-diastolic pressure, *EDPVR* end-diastolic pressure-volume relationship, *EDV* end-diastolic volume, *EF* ejection fraction, *LV* left ventricle, *SV* stroke volume, *RV* right ventricle.

* p<0.05 versus controls.

### 3.2 Correlation of CMR imaging biomarkers against EDPVR slope

**Fig. 1:**
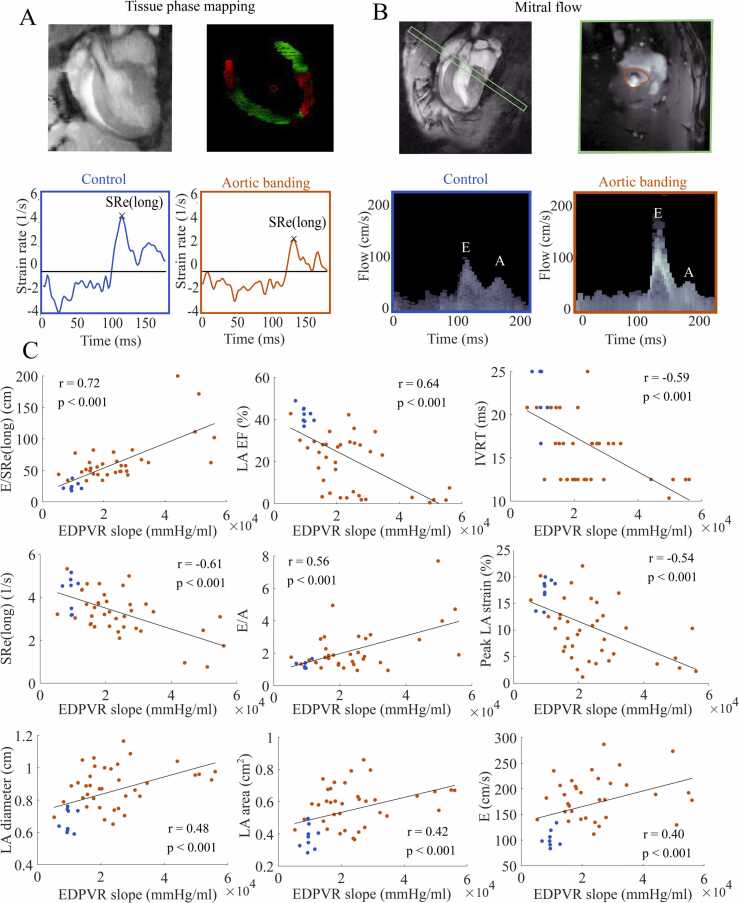
**Correlation of CMR imaging biomarkers against EDPVR slope. (A):*****Top left:*** Representative cine cardiovascular magnetic resonance (CMR) images of the long-axis view of the heart. ***Top right:*** Velocity encoding data from the left ventricle in the same projection. ***Bottom:*** Representative left ventricular longitudinal strain rate curves from control (left) and aortic banding (right). **(B):** CMR based mitral flow velocity measurement. ***Top panels:*** Region of interest definition for mitral flow velocity measurement. ***Bottom panels:*** Mitral flow velocity from ***left:*** Control animals, ***right:*** Aortic banded animals. **(C):** Correlation of CMR imaging biomarkers against the slope of the end-diastolic pressure-volume relationship (EDPVR, mmHg/ml). Correlation plots including controls (n=8–9, blue symbols) and aortic banded (n=34–38, orange symbols) rats with regression line (black), and where r is the value of Pearson’s correlation coefficient. Due to low temporal resolution with CMR, IVRT appears discontinuous. *EF* ejection fraction, *IVRT* isovolumetric relaxation time, *LA* left atrial, *SR* strain rate, *SRe(long)* peak early diastolic longitudinal strain rate

The CMR imaging biomarkers E/SRe(long), LA EF, IVRT, SRe(long), E/A, peak LA strain, LA diameter, LA area, and E all demonstrated correlation to the EDPVR slope (|r|=0.4–0.72, Fig. 1, Table 2). E/SRe(long) demonstrated the strongest correlation to the EDPVR slope.

**Table 2:** Analysis of CMR imaging biomarkers against the EDPVR slope.

	**Person’s correlation**		**Multivariable (pre stepwise regression)**		**Multivariable (post stepwise regression)**	
**Imaging biomarkers**	**r**	**p-value**	**β**	**p-value**	**β**	**p-value**
**E/SRe(long)**	0.72	<0.001	101	0.261	185	<0.001
**LA EF**	−0.64	<0.001	−34889	0.124	−29210	0.015
**SRe(long)**	−0.61	<0.001	−1973	0.457		
**IVRT**	−0.59	<0.001	−596	0.289		
**E/A**	0.56	<0.001	1447	0.320		
**Peak LA strain**	−0.54	<0.001	263	0.612		
**LA diameter**	0.48	<0.001	14958	0.676		
**LA area**	0.42	<0.001	−36774	0.270		
**E**	0.40	<0.001	24	0.623		

Pearson’s correlation and multivariate analysis of cardiovascular magnetic resonance (CMR) imaging biomarkers in predicting the slope of the end-diastolic pressure-volume relationship (EDPVR). Data presented are Pearson’s correlation coefficient, r, estimated coefficient from multiple linear regression, β, and corresponding p-values.

*EF* ejection fraction, *IVRT* isovolumetric relaxation time, *LA* left atrial, *SR* strain rate, *SRe(long)* peak early diastolic longitudinal strain rate, *E* early mitral inflow velocity, *E/A* early-to-late mitral inflow velocity ratio.

### 3.3 Correlation of echocardiographic imaging biomarkers against EDPVR slope

**Fig. 2:**
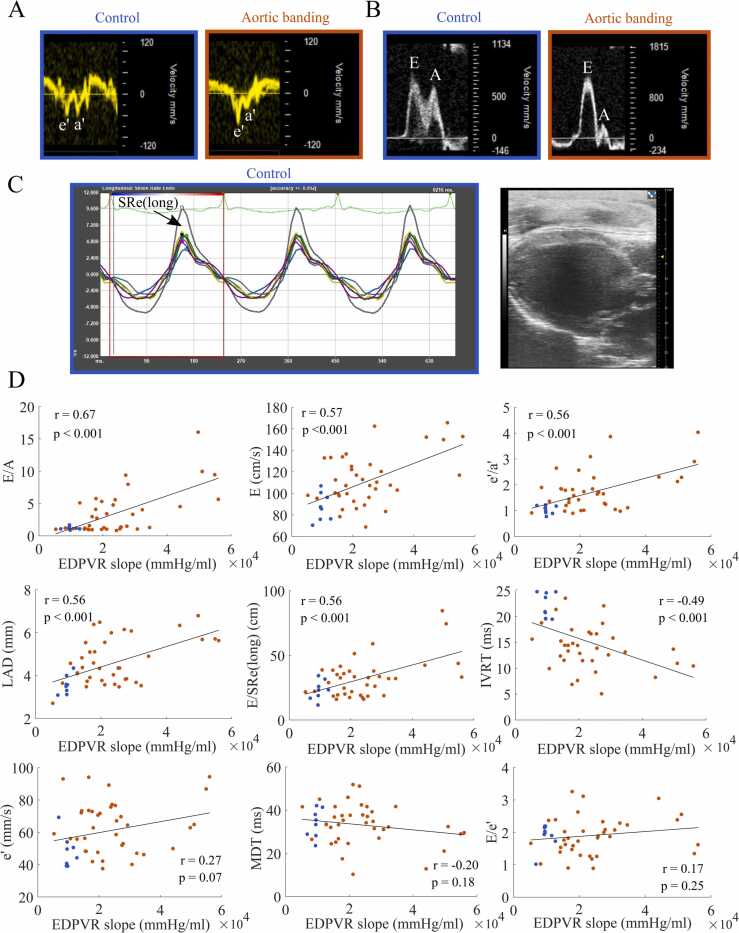
**Correlation of echocardiographic imaging biomarkers against EDPVR slope. (A):** Representative traces of echocardiography tissue Doppler based early LV annular velocity from ***left:*** Control animal, ***right:*** Aortic banded animal. (**B):** Representative traces of echocardiography PW Doppler based LV mitral inflow velocity from ***left:*** Control animal, ***right***: Aortic banded animal. **(C):*****Left:*** Representative trace from echocardiography based, long-axis speckle tracking demonstrating peak early diastole longitudinal strain rate (SRe(long)). ***Right:*** B-mode long-axis image from the same animal. **(D):** Correlation of echocardiographic imaging biomarkers against the slope of the end-diastolic pressure-volume relationship (EDPVR, mmHg/ml) plots including controls (n=8–9, blue symbols) and aortic banded rats (n=35–38, orange symbols) with regression line (black). *MDT* mitral E-wave deceleration time, *IVRT* isovolumetric relaxation time, *LA* left atrial, *SR* strain rate, *PW* pulsed wave.

The echocardiographic imaging biomarkers E/A, E, LA diameter, e’/a’, E/SRe(long), and IVRT demonstrated correlation to the EDPVR slope (|r|=0.49–0.67), while no statistically significant correlation was found between the EDPVR slope and e’, MDT and E/e’ (Fig. 2, Table 3). E/A demonstrated the strongest correlation to the EDPVR slope.

**Table 3:** Analysis of echocardiographic imaging biomarkers against the EDPVR slope.

	**Person’s correlation**		**Multivariable (pre stepwise regression)**		**Multivariable (post stepwise regression)**	
**Imaging biomarkers**	**r**	**p-value**	**β**	**p-value**	**β**	**p-value**
**E/A**	0.67	<0.001	1432	0.051	1697	<0.001
**E**	0.57	<0.001	−67	0.487		
**LA diameter**	0.56	<0.001	−505	0.801		
**e’/a’**	0.56	<0.001	5815	0.009	4798	0.014
**E/SRe(long)**	0.56	<0.001	199	0.155		
**IVRT**	−0.49	<0.001	−728	0.053	−705	0.011
**e’**	0.27	0.074				
**MDT**	−0.20	0.175				
**E/e’**	0.17	0.250				

Pearson’s correlation and multivariate analysis of echocardiographic imaging biomarkers in predicting the slope of the end-diastolic pressure-volume relationship (EDPVR). Data presented are Pearson’s correlation coefficient, r, estimated coefficient from multiple linear regression, β, and belonging p-values.

*MDT* mitral E-wave deceleration time, *IVRT* isovolumetric relaxation time, *LA* left atrial, *SR* strain rate, *E/A* early-to-late mitral inflow velocity ratio, *e'/a'* early-to-late LV annular velocity ratio, *SRe(long)* peak early diastolic longitudinal strain rate.

### 3.4 Multivariate analysis

**Fig. 3:**
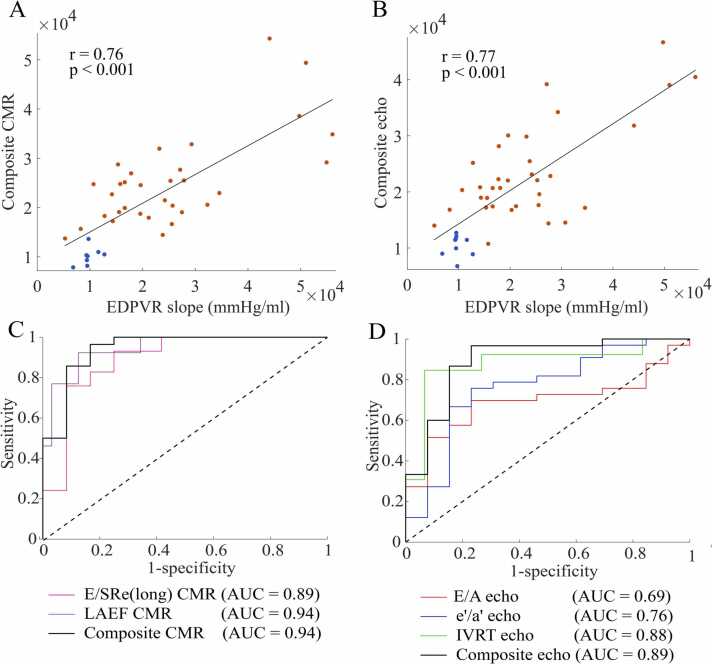
**Analysis of composite parameters.** Correlation plots of slope of the end-diastolic pressure-volume relationship (EDPVR, mmHg/ml) against composite parameters from **(A):** Echocardiographic and **(B):** Cardiovascular magnetic resonance (CMR) imaging biomarkers. The Pearson’s correlation coefficient (r) and p-values are shown on the graphs together with controls (n=8, blue symbols), aortic banding rats (n=35/32, orange symbols) and regression line (black). Receiver operating characteristics curve for predicting increased EDPVR slope of composite parameters from **(C):** Echocardiography and **(D):** CMR, and the individual imaging biomarkers included in the composites with area under the curve (AUC). *IVRT* isovolumetric relaxation time, *EF* ejection fraction, *LA* left atrial, *SR* strain rate.

A multivariate stepwise regression was performed to simplify the multivariate model to include only image biomarkers with independent predictive value (independent predictors). Imaging biomarkers with a statistically significant correlation to the EDPVR slope were included in the multivariate regression analysis. Results for CMR identified E/SRe(long) and LA EF as independent predictors of the EDPVR slope (Table 2). From echocardiography, E/A, e’/a’, and IVRT were left as independent predictors (Table 3). Composite parameters from both CMR and echocardiography showed strong correlation with the EDPVR slope (r=0.76 and r=0.77, respectively, Fig. 3A-B).

### 3.5 Receiver operating characteristic analysis

Based on the normal range of the EDPVR slope defined by controls, we investigated the ability of CMR and echocardiographic imaging biomarkers, along with composite scores, to predict increased EDPVR slope (Fig. 3C-D). The findings of the receiver operating characteristic analysis indicated that CMR imaging biomarkers in general performed better than echocardiographic imaging biomarkers (CMR: AUC=0.76–0.94; echocardiography: AUC=0.55–0.82), where eight of out the ten highest AUC values were CMR imaging biomarkers (Table 4). The composite parameter of CMR also demonstrated better performance than the composite parameter from echocardiography (CMR: AUC=0.94; echocardiography: AUC = 0.89). Particularly imaging biomarkers related to the left atrium such as LA EF and LA strain demonstrated strong predictive value of increased EDPVR slope.

**Table 4:** Results from receiver operating characteristic analysis.

**CMR imaging biomarkers**	**Echocardiographic imaging biomarkers**	**AUC (CI)**
**LA EF**		0.94 (0.88−1.00)
**E/SRe(long)**		0.89 (0.79−0.99)
	**IVRT**	0.88 (0.78−0.98)
**Peak LA strain**		0.88 (0.78−0.98)
**IVRT**		0.88 (0.77−0.98)
**LA diameter**		0.86 (0.76−0.97)
**LA area**		0.86 (0.75−0.97)
	**LA diameter**	0.82 (0.69−0.94)
**E**		0.80 (0.66−0.94)
**SRe(long)**		0.78 (0.64−0.91)
**E/A**		0.76 (0.62−0.91)
	**e’/a’**	0.76 (0.62−0.90)
	**E**	0.72 (0.57−0.88)
	**e’**	0.70 (0.54−0.86)
	**E/A**	0.69 (0.53−0.85)
	**E/SRe(long)**	0.65 (0.48−0.82)
	**MDT**	0.55 (0.37−0.73)
	**E/e'**	0.55 (0.36−0.73)

Area under the curve (AUC) with 95% confidence interval (CI) for imaging biomarkers in predicting increased slope of the end-diastolic pressure-volume relationship (EDPVR) using cardiovascular magnetic resonance (CMR) and echocardiography.

*EF* ejection fraction, *MDT* mitral E-wave deceleration time, *IVRT* isovolumetric relaxation time, *LA* left atrial, *SR* strain rate, *E/A* early-to-late mitral inflow velocity ratio, *e'/a*' early-to-late LV annular velocity ratio, *SRe(long)* peak early diastolic longitudinal strain rate.

### 3.6 Native T1 mapping

**Fig. 4:**
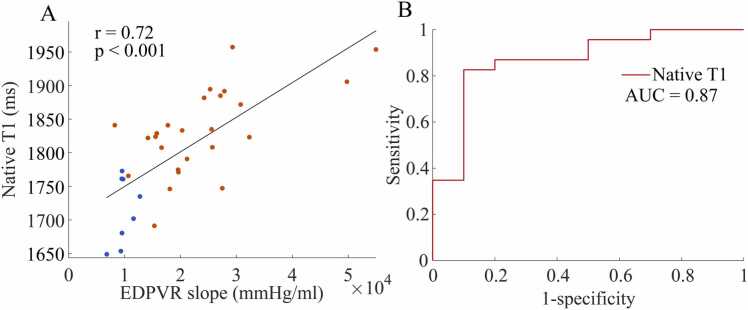
**Analysis of native T1 values.** Analysis of native T1 values in predicting the slope of the end-diastolic pressure-volume relationship (EDPVR, mmHg/ml). **(A):** Correlation plot of native T1 values against the EDPVR slope. The Pearson’s correlation coefficient (r) and p-values are shown on the graph together with data points of controls (n=8, blue symbols) and aortic banding (n=25, orange symbols) and regression line (black). **(B):** receiver operating characteristic curve of native T1 values in predicting increased EDPVR slope. Results represented as area under the curve (AUC).

We achieved high-quality native T1 mapping in 8 controls and 25 aortic banded rats. Pearson’s correlation analysis (Fig. 4A) demonstrated strong correlation between native T1 values and the EDPVR slope (r=0.72). Receiver operating characteristic analysis (Fig. 4B) indicated good performance of native T1 values in predicting increased EDPVR slope (AUC=0.87).

## 4 Discussion

In the current study we aimed at systematically evaluate how well commonly used imaging biomarkers correlate to cardiac stiffness. We demonstrate how several parameters commonly employed in preclinical studies have poor ability to predict cardiac chamber stiffness in a pressure-overload induced model of diastolic dysfunction. Furthermore, we identified several imaging biomarkers from CMR and echocardiography, including E/SRe(long), E/A and LA EF, that were able to predict cardiac chamber stiffness and demonstrated the value of several promising imaging biomarkers of diastolic dysfunction.

Few previous studies have investigated how imaging biomarkers reflect changes in the EDPVR slope. However, one study in humans found that native T1 values were correlated with the EDPVR slope comparably to the extracellular volume (native T1 values, r=0.54; extracellular volume fraction, r=0.50) [14]. EDPVR can also be estimated using a single-beat estimation developed by Klotz et al. [12]. One study used this estimation of EDPVR to divide patients with severe aortic stenosis into low and high chamber stiffness, finding the imaging biomarkers LA volume, MDT, septal e’ and E/e’ to be statistically different in the two groups [2]. Another study used this estimation of EDPVR in patients with heart failure with preserved ejection fraction and found higher LV EF to be associated with increased EDPVR slope in these patients [8].

### 4.1 Left atrial imaging biomarkers

We found strong performance of LA imaging biomarkers in both correlation with the EDPVR slope and in prediction of increased EDPVR slope. This is in line with previous studies finding that LA size is used to identify diastolic dysfunction both in humans [15], [32] and mice [29], and known to reflect the average effect of alteration to the LV filling pressures over time [33]. In recent years, there has been more attention towards LA function, such as LA strain and LA EF, as early markers of diastolic dysfunction [34]. This highlights the physiological importance of LA function as gauge of downstream ventricular stiffness, reflecting not only cumulative impact of filling abnormalities but also early changes in diastolic dysfunction. We found LA EF to have one of the strongest correlations with the EDPVR slope and the highest AUC of all investigated imaging biomarkers, indicating that these parameters have value also in rodents.

### 4.2 E, E/A and MDT

The physiological relevance of mitral filling biomarkers lies in their ability to reflect alterations in LV compliance and relaxation [35]. Imaging biomarkers E and E/A- demonstrated low to moderate correlations with the EDPVR slope in both CMR and echocardiography. In humans, E/A tends to decrease in early stages of diastolic dysfunction before increasing in more severe cases, a phenomenon called pseudo-normalization [15]. In our study, visual inspection of the correlation plot did not reveal any decrease in E/A.

We did not observe any statistically significant correlation between MDT and the EDPVR slope. In humans, MDT is often shortened in cases of elevated filling pressure and severe diastolic dysfunction. However, it can be prolonged in situations where there is a high E-peak [15]. It is important to note that precise measurements of MDT can be challenging in rodents due to their high heart rate, which can result in merger of the declining slope of the E-wave with the rising A-wave. This could contribute to the observed lack of utility. From this, our findings suggest that MDT is not a reliable indicator of increased EDPVR slope in rats.

### 4.3 e’, E/e’ and e’/a’

Ventricular deformation velocities are physiologically significant as they reflect the interplay between relaxation, restoring forces and chamber stiffness [36]. The echocardiographic imaging biomarkers e’ and E/e’ have demonstrated their value in assessing diastolic dysfunction in humans and are incorporated in clinical guidelines for assessing and grading diastolic dysfunction [15]. However, measurement of longitudinal e’ in rodents is challenging compared to in humans due to differences in thoracic geometry. Excessive pressure on the thorax or placing the animal in a prone and tilted position is often needed for adequate acoustic window, both of which can significantly affect hemodynamics and compromise the standardization of the recordings. An alternative strategy, which was used in this study, is to record e’ in the radial direction with the sampling volume on the posterior wall.

Our results revealed no correlation between e’ and E/e’ when compared to the EDPVR slope. This lack of correlation might be related to the fact that loss of global longitudinal function has shown to be an earlier marker of cardiac dysfunction than loss in global circumferential and radial function [37]. Furthermore, a previous modelling study has demonstrated that e’ can increase with faster early relaxation [36]. In our study, we observed a decreased tau in aortic banded rats in comparison to controls, which may be explained by higher restoring forces due to concentric hypertrophy combined with an enhanced Serca2 function previously shown in aortic banded rats [38]. The decreased tau indicates faster relaxation, which could also contribute to a higher e’. These findings underscore the nuance of using e’ as an indicator of diastolic dysfunction in rodent models.

We found e’/a’ to be one of the best performing echocardiographic imaging biomarkers for predicting the EDPVR slope. Notably, this observation occurs without statistically significant alterations in e’, suggesting that the increase in e’/a’ in aortic banded rats mainly is driven by a reduction in a’. Our study revealed reduced LA EF and LA strain, which potentially implies that impaired atrial function underlies the reduction in a’. Increased LV chamber stiffness could also be a contributor to reductions in a’ by opposing myocardial deformation in late diastole. Interestingly, in a rat model with high grade systemic inflammation and increased cardiac fibrosis, it has been shown that e’ and e’/a’ can be reduced [39], which shows the importance of acknowledging that e’/a’ can vary depending on aetiology and acquisition method.

### 4.4 SRe(long) and E/SRe(long)

Ventricular diastolic strain rates reflect intrinsic myocardial relaxation and ventricular compliance [40]. In our study, we observed a moderate correlation between SRe(long) and the EDPVR slope. SRe can be measured in the longitudinal direction using both CMR and echocardiography, similar to the direction of e’ in humans. Of all the imaging biomarkers tested, E/SRe(long) from CMR demonstrated the strongest correlation to the EDPVR slope. Also, from echocardiography E/SRe(long) measured in long-axis demonstrated moderate correlation to the EDPVR slope and could therefore be a good alternative to E/e’ in rodents.

### 4.5 IVRT

Both CMR and echocardiographic measurements of IVRT demonstrated correlation with the EDPVR slope. Interestingly, our findings revealed a shortening of IVRT with increasing EDPVR slope. A shorter IVRT is also reported in another mice study of aortic constriction [41], while in a mouse model of aortic banding reported by Schnelle et al. IVRT was found to be prolonged in diseased mice [16]. Several factors may contribute to a reduction in IVRT in this study, including faster relaxation and increased LA pressure that shortens the time to mitral valve opening [42]. As IVRT is found to both decrease and increase in similar rodent models of aortic banding [16], caution should be exercised when using IVRT as a sole imaging biomarker to exclude diastolic dysfunction in rodents.

### 4.6 Native T1 mapping

T1 mapping provides a metric for diffuse myocardial fibrosis which is a key determinant of passive myocardial stiffness, allowing linking tissue composition with ventricular compliance and diastolic function [43]. Native T1 value was one of the imaging biomarkers that exhibited the strongest correlation with EDPVR slope, however, not of the highest AUC in predicting increased EDPVR slope. The performance of native T1 values may be related to its ability to estimate extent of diffuse myocardial fibrosis [43], which substantially contributes to the passive myocardial stiffness. Although the accuracy may be superior, technical limitations hinder the acquisition of high quality native T1 mapping. Particularly, ECG triggering can pose challenges in animals with poor ECG signals [44], [45], and animals afflicted by disease may be unable to tolerate the duration of anaesthesia needed for image acquisition. Overall, our findings demonstrate the great potential of native T1 values to predict the EDPVR slope. However, further research is needed in rodents to address the current technical limitation in acquisition.

### 4.7 Comparison between CMR and echocardiography

Both CMR and echocardiographic imaging biomarkers correlated with LV chamber stiffness. In general, individual imaging biomarkers obtained from CMR predicted the EDPVR slope better than those obtained from echocardiography, both in observed correlations with linear regression and in AUC for identifying increased EDPVR slope. This demonstrates the value and potential of CMR as a tool to evaluate diastolic function in rodents, beyond its current use to assess systolic function and geometrical changes [29].

Better performance of CMR may be explained by several factors, amongst them the challenge in obtaining optimal acoustic windows in rats and the higher inter-observer variability in echocardiographic recordings compared to CMR [44]. Furthermore, several imaging biomarkers available from CMR are not feasible with echocardiography in rodents, such as native T1 mapping [45]. However, combining echocardiographic markers into a composite score yields performance at a similar level as CMR. These findings highlight the importance of utilizing several markers for the measurement of diastolic function. While the specific choice and weighting of each imaging biomarker included in a composite score may vary for different species and aetiologies, our results emphasize the potential value of composite scores. Further research with external validation in separate cohorts to avoid overfitting, and exploration of composite score performance in different pathophysiological states.

### 4.8 Implications for assessment of diastolic dysfunction

An implication of the results from this study is to the validation of diastolic dysfunction. Guidelines exist for echocardiographic measurement of diastolic function in rodents [28], but these guidelines are based on validation towards LV end-diastolic pressure (LVEDP) [46]. As LVEDP does not take cardiac chamber volume into account, it is unable to discern diastolic dysfunction from altered loading conditions [47]. Studies reporting on diastolic function in rats commonly use these imaging biomarkers to rule out diastolic dysfunction, but LVEDP do not reflect the true chamber stiffness and animals with increased chamber stiffness can therefore wrongfully be categorised as having normal diastolic function. The results of this study can therefore provide new insight into which imaging biomarkers are related to chamber stiffness, and thereby facilitate these studies.

## 5 Limitations

A limitation of this study is that we included only male rats due to lack of an establishment of the aortic constriction model using O-rings in female rats. It is important to acknowledge that imaging biomarkers may exhibit variations in response across different species, genders [48], and disease models. Potential differences in biomarkers response between genders should be examined, thus, future investigations addressing these aspects are needed to offer additional and important insights into the performance of imaging biomarkers in predicting LV chamber stiffness.

Other indices of LV stiffness and impaired filling such as the passive myocardial elastic properties reflected by the stress-strain relationship or LV filling pressure would also be valuable to explore other aspects of LV stiffness [10]. In this study, native T1 was investigated but other indices of the extracellular matrix composition would also be interesting to explore, such as extracellular volume, T2, T2* and T1rho mapping [45].

Conducting speckle tracking echocardiography in rodents and obtaining recordings that include the apical tip is not always feasible. Nonetheless, meaningful information could still be derived from the rest of the myocardium, although this could give rise to variation between recordings.

Our findings highlight the strong performance of imaging biomarkers related to the left atrium in predicting the EDPVR slope. However, among the echocardiographic imaging biomarkers investigated, LA diameter was the only imaging biomarker measured from the left atrium. Therefore, future investigations should incorporate standardized recordings of the left atrium to evaluate imaging biomarkers such as LA area, LA EF and LA strain, and investigate whether these imaging biomarkers outperform those investigated in this study.

## 6 Conclusions

In this paper, we show that several imaging biomarkers of diastolic function from CMR and echocardiography correlate with LV chamber stiffness as measured by the EDPVR slope in a rat model of pressure overload. Our findings highlight the CMR imaging biomarkers native T1 mapping and E/SRe(long) and the echocardiographic imaging biomarker E/A demonstrated the most effective parameters for assessing LV chamber stiffness. A composite score of echocardiographic imaging biomarkers demonstrated comparable predictive value to CMR. These findings provide valuable insights into how imaging biomarkers can serve as non-invasive tools for reflecting changes in LV chamber stiffness. To refine the utility of imaging biomarkers in assessing LV chamber stiffness, future investigations should investigate species and model-specific variations.

## Funding

This study was supported by the KG Jebsen Center for Cardiac Research (Oslo, Norway), The South-Eastern Norway Regional Health Authority (Oslo, Norway) [grant number: 2022013], Familien Blix’ Fond Til Fremme Av Medisinsk Forskning (Oslo, Norway), Olav Raagholt og Gerd Meidel Raagholts stiftelse for forskning (Oslo, Norway).

## CRediT authorship contribution statement

**Espe Emil Knut Stenersen:** Writing – review & editing, Writing – original draft, Validation, Supervision, Software, Resources, Methodology, Investigation, Funding acquisition, Conceptualization. **Hauge-Iversen Ida Marie:** Writing – review & editing, Writing – original draft, Visualization, Software, Project administration, Methodology, Investigation, Formal analysis, Data curation, Conceptualization. **Nordén Einar S:** Writing – review & editing, Writing – original draft, Methodology, Investigation, Conceptualization. **Zhang Lili:** Writing – review & editing, Software, Formal analysis. **Sjaastad Ivar:** Writing – review & editing, Writing – original draft, Resources, Methodology, Investigation, Funding acquisition, Conceptualization. **Melleby Arne Olav:** Writing – review & editing, Investigation. **Espeland Linn:** Writing – review & editing, Investigation.

## Declaration of Competing Interest

The authors declare that they have no known competing financial interests or personal relationships that could have appeared to influence the work reported in this paper.

## Data Availability

A complete dataset is publicly available at: https://doi.org/10.5281/zenodo.14801836.
