# Supplementary material for: Non-invasive estimation of left ventricular chamber stiffness using cardiovascular magnetic resonance and echocardiography
Source: J Cardiovasc Magn Reson. 2025 Jan 31;27(1):101849. doi: 10.1016/j.jocmr.2025.101849 (PMC12182815; doi:10.1016/j.jocmr.2025.101849)
Supplement: Supplementary file 1 — Supplementary material [file mmc1.docx]

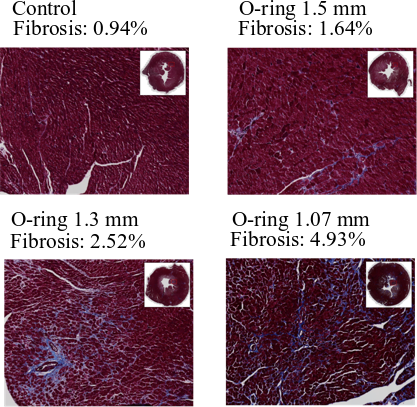
Supplementary Results:

**Supplementary Figure 1:** Representative images from mid-left ventricular sections stained for collagen with Masson’s Trichrome at 12 weeks after aortic banding or sham operation. Upper left: sham operated control, upper right: O-ring inner diameter 1.5 mm, lower left: O-ring inner diameter 1.3 mm and lower right: O-ring inner diameter 1.07.

|  | n | Control | O-ring 1.5 mm | O-ring 1.3 mm | O-ring 1.07 mm |
| --- | --- | --- | --- | --- | --- |
| E/SRe(long) (cm) | 8/6/23/4 | 22.5 (21.0-25.2) | 47.1 (44.0-60.1) | 53.4 (46.3-62.4)* | 106.6 (91.0-126.2)* |
| LA EF (%) | 9/6/26/4 | 42.5 (39.6-44.9) | 29.4 (28.3-33.0) | 18.3 (3.4-27.6)* | 4.6 (1.5-12.8)* |
| IVRT (ms) | 9/6/26/4 | 24.5 (21.0-25.2) | 20.8 (17.7-20.8) | 16.7 (12.5-16-7)* | 12.5 (12.0-13.5)* |
| SRe(long) (1/s) | 9/6/27/4 | 4.52 (3.48-4.64) | 3.50 (2.62-4.19) | 3.21 (2.75-3.72) | 2.10 (1.49-2.62)* |
| E/A | 8/6/23/4 | 1.38 (1.14-1.41) | 1.36 (1.15-1.42) | 1.86 (1.61-2.86) | 3.52 (2.76-4.91)*† |
| Peak LA strain (%) | 9/6/27/4 | 18.28 (16.71-18.74) | 14.44 (11.36-16.71) | 8.44 (5.17-12.37)* | 3.80 (2.72-6.49)* |
| LA diameter (mm) | 9/6/27/4 | 6.39 (6.14-7.34) | 7.76 (6.93-8.22) | 8.88 (8.18-10.05)* | 9.58 (9.47-9.64)* |
| LA area (mm^2^) | 9/6/27/4 | 38.2 (32.7-40.5) | 45.0 (38.4-51.9) | 60.3 (51.6-67.9)* | 63.9 (59.8-66.5)* |
| E (cm/s) | 8/6/23/4 | 98 (92-109) | 149 (137-194) | 189 (161-209)* | 178 (165-202) |

**Supplementary Table 1:** Median value (interquartile range) for cardiovascular magnetic resonance imaging biomarker for sham operated control, and O-rings with inner diameters of 1.5 mm, 1.3 mm and 1.07 mm. EF: Ejection fraction, IVRT: Isovolumetric relaxation time, LA: Left atrial, SRe(long): Peak early diastolic longitudinal strain rate.

|  | n | Control | O-ring 1.5 mm | O-ring 1.3 mm | O-ring 1.07 mm |
| --- | --- | --- | --- | --- | --- |
| E/A | 9/6/27/4 | 1.18 (1.08-1.22) | 2.62 (1.08-4.84) | 1.49 (1.07-4.32) | 7.81 (4.58-11.48)* |
| E (cm/s) | 9/6/27/4 | 86.8 (76.4-96.2) | 98.4 (86.7-111.3) | 107.2 (98.0-121.2)* | 151.4 (144.2-156.0)* |
| e’/a’ | 9/6/27/4 | 1.10 (0.93-1.18) | 1.84 (1.22-2.17) | 1.58 (1.23-1.83)* | 2.21 (2.02-2.73)* |
| LAD (mm) | 9/6/27/4 | 3.58 (3.31-4.01) | 3.82 (3.58-4.11) | 4.59 (3.81-5.63)* | 5.66 (5.31-5.96)* |
| E/SRe(long) (cm) | 8/6/27/4 | 23.2 (18.4-27.4) | 23.7 (17.5-31.2) | 32.1 (20.9-38.2) | 53.8 (33.1-77.0)* |
| IVRT (ms) | 9/5/25/4 | 21.0 (19.6-24.6) | 15.3 (11.4-16.7) | 14.4 (11.6-16.7)* | 12.3 (10.8-13.7)* |
| e’ (mm/s) | 9/6/27/4 | 44.2 (39.6-50.6) | 64.5 (49.2-75.1) | 59.1 (49.1-72.5) | 66.0 (64.3-73.9)* |
| MDT (ms) | 9/6/27/4 | 33.5 (28.9-38.0) | 35.6 (32.5-41.3) | 34.2 (26.9-40.4) | 31.0 (27.4-33.6) |
| E/e’ | 9/6/27/4 | 1.98 (1.90-2.15) | 1.79 (1.27-2.22) | 1.82 (1.43-2.16) | 2.14 (1.82-2.43) |

**Supplementary Table 2:** Median value (interquartile range) for echocardiographic imaging biomarker for sham operated control, and O-rings with inner diameters of 1.5 mm, 1.3 mm and 1.07 mm. IVRT: Isovolumetric relaxation time, LAD: Left atrial diameter, MDT: Mitral E-wave deceleration time, SRe(long): Peak early diastolic longitudinal strain rate.


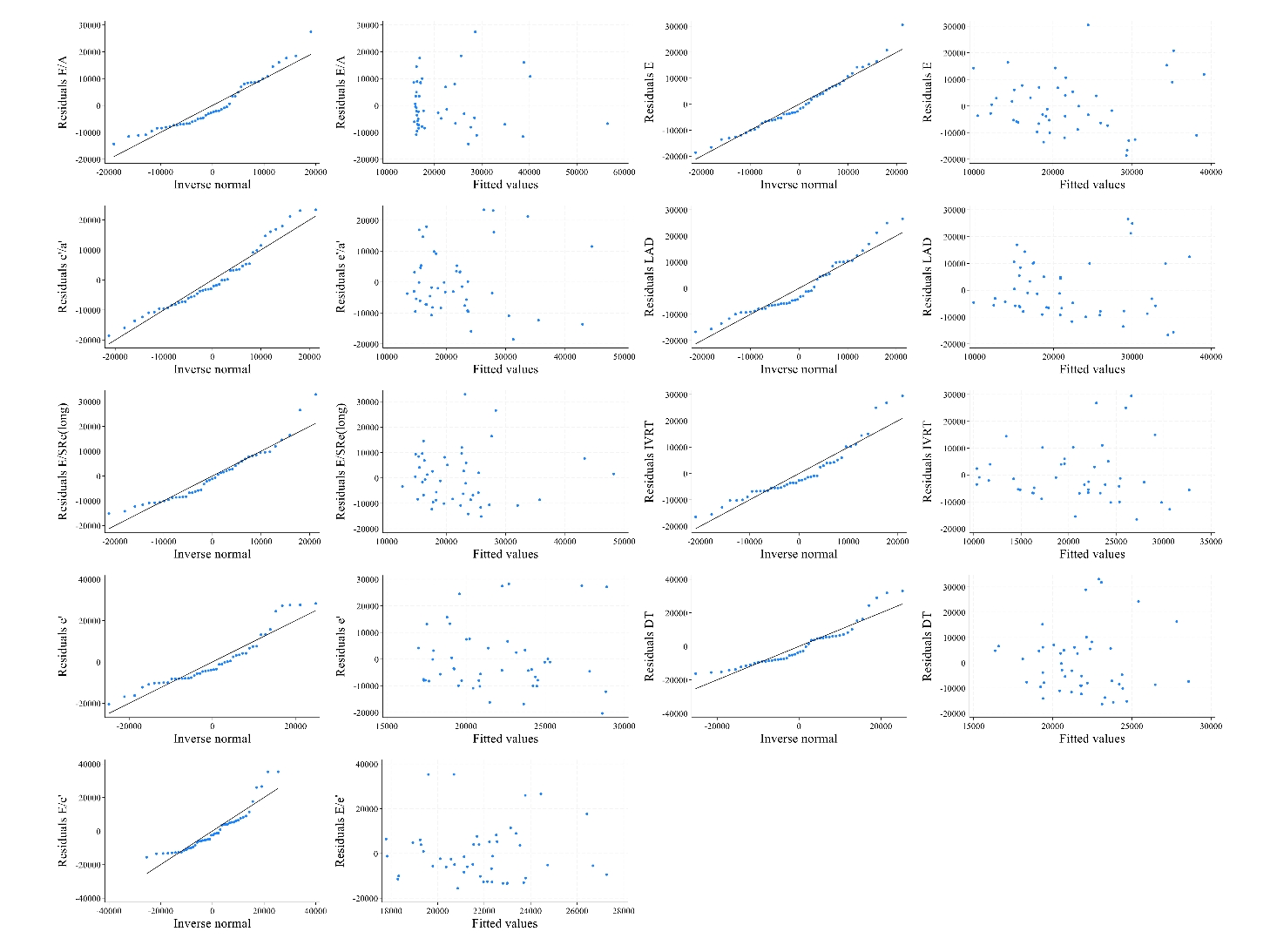
Normality and variance of residuals from linear regression was checked for all imaging biomarkers from echocardiography (Supplementary Figure 2) and CMR (Supplementary Figure 3). Normal distribution was checked using quantile-quantile plots (QQ-plots) and variance of residuals were checked using plotting the residuals against the observed values.

**Supplementary Figure 2**: Normal distribution from QQ-plot and variance of residuals of echocardiographic imaging biomarkers against the predicted fitted value.


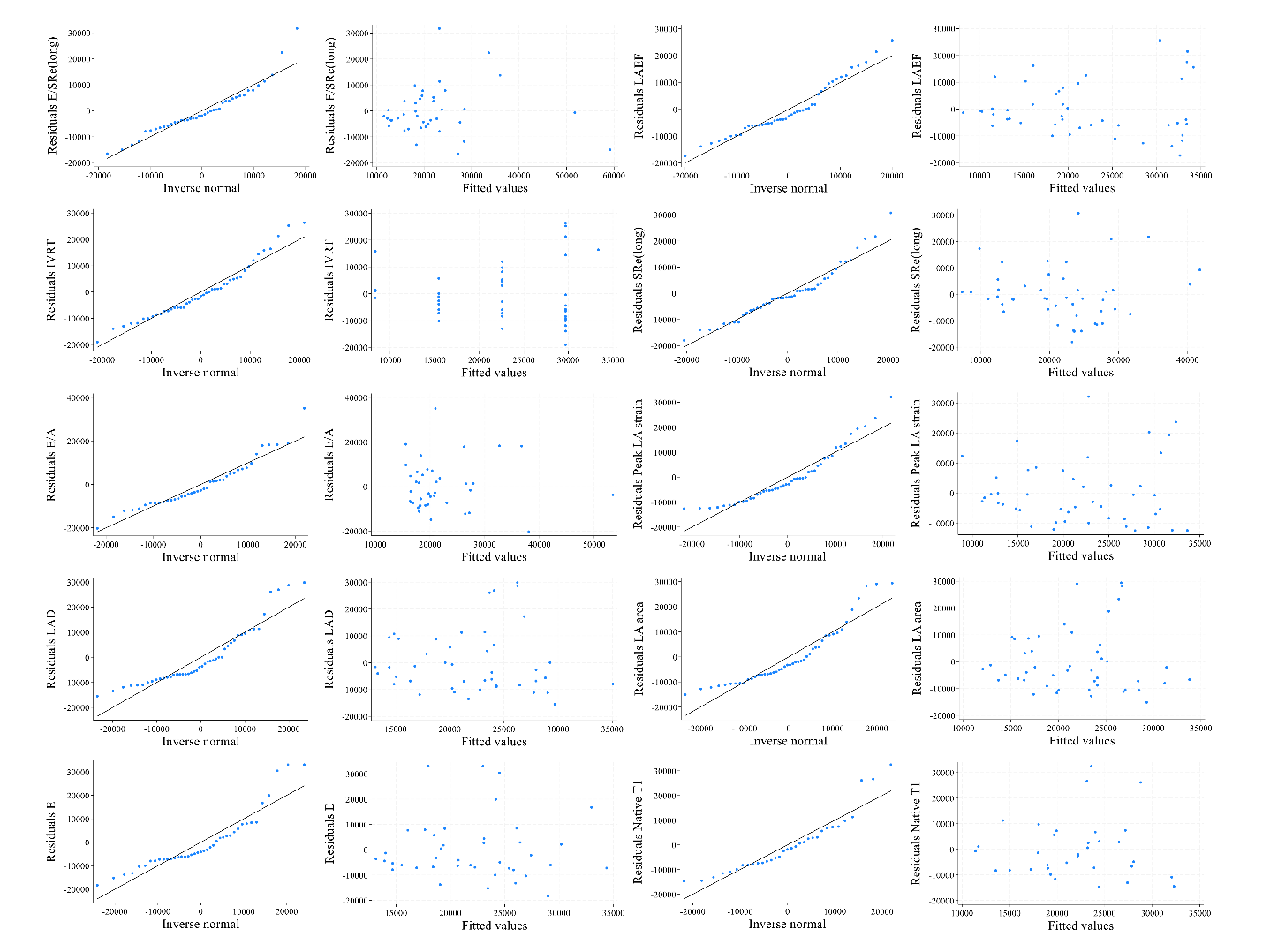
**Supplementary Figure 3**: Normal distribution from QQ-plot and variance of residuals of cardiovascular magnetic resonance imaging biomarkers against the predicted fitted value.
